# Globalization and social determinants of health: Introduction and methodological background (part 1 of 3)

**DOI:** 10.1186/1744-8603-3-5

**Published:** 2007-06-19

**Authors:** Ronald Labonté, Ted Schrecker

**Affiliations:** 1 Department of Epidemiology and Community Medicine, Faculty of Medicine and Institute of Population Health, University of Ottawa, Canada

## Abstract

Globalization is a key context for the study of social determinants of health (SDH). Broadly stated, SDH are the conditions in which people live and work, and that affect their opportunities to lead healthy lives.

In this first article of a three-part series, we describe the origins of the series in work conducted for the Globalization Knowledge Network of the World Health Organization's Commission on Social Determinants of Health and in the Commission's specific concern with health equity. We explain our rationale for defining globalization with reference to the emergence of a global marketplace, and the economic and political choices that have facilitated that emergence. We identify a number of conceptual milestones in studying the relation between globalization and SDH over the period 1987–2005, and then show that because globalization comprises multiple, interacting policy dynamics, reliance on evidence from multiple disciplines (transdisciplinarity) and research methodologies is required. So, too, is explicit recognition of the uncertainties associated with linking globalization – the quintessential "upstream" variable – with changes in SDH and in health outcomes.

## Background: health equity and the social determinants of health

This article is the first in a series of three that together describe research strategies to address the relation between contemporary globalization and the social determinants of health (SDH) through an 'equity lens,' and invite dialogue and debate about preliminary findings. The global commitment to health equity is not new; in 1978, the landmark United Nations conference in Alma-Ata declared the goal of health for all by the year 2000 [1]. Yet in 2007, despite progress toward that goal, millions of people die or are disabled each year from causes that are easily preventable or treatable [2]. Recent reviews [3,4] of research on HIV/AIDS, tuberculosis and malaria, communicable diseases that together account for almost six million deaths per year, identify poverty, gender inequality, development policy and health sector 'reforms' that involve user fees and reduced access to care as contributors. More than 10 million children under the age of five die each year, "almost all in low-income countries or poor areas of middle-income countries" [5](p. 65; see also [6]) and from causes of death that are rare in the industrialized world. Undernutrition – an unequivocally economic phenomenon, resulting from inadequate access to the resources for producing food or the income for purchasing it – is an underlying cause of roughly half these deaths [6], and lack of access to safe water and sanitation contributes to 1.5 million [7]. An expanding body of literature describes a similarly unequal distribution of many non-communicable diseases and injuries, with incidence and vulnerability often directly related to poverty, economic insecurity or economic marginalization [8-15]. Three decades of rapid global market integration have occurred in parallel with these trends; these articles address the relation between these two patterns.

Our work follows a trajectory of inquiry initiated by the World Health Organization (WHO). In 2001, the WHO Commission on Macroeconomics and Health turned much conventional wisdom on its head by demonstrating that health is not only a benefit of development, but also is indispensable to development [16]. Illness all too often leads to "medical poverty traps" [17], creating a vicious circle of poor nutrition, forgone education, and still more illness – all of which undermine the economic growth that is necessary, although not sufficient, for widespread improvements in health status. Like the earlier Alma-Ata commitment to health for all, most of the Commission's recommendations, which it estimated could have saved millions of lives each year by the end of the current decade, have not been translated into policy. Further, the Commission did not inquire into how the economic and geopolitical dynamics of a changing international environment ('globalization') support and undermine health, or how these dynamics can be channelled to improve population health.

In 2005, WHO established the Commission on Social Determinants of Health (CSDH), on the premise that action on SDH is the fairest and most effective way to improve health for all people and reduce inequalities. Central to the Commission's remit is the promotion of health equity, which is defined in the literature as "the absence of disparities in health (and in its key social determinants) that are systematically associated with social advantage/disadvantage" [18](p. 256). Social determinants of health, broadly stated, are the conditions in which people live and work that affect their opportunities to lead healthy lives. Good medical care is vital, but unless the root social causes that undermine people's health are addressed, the opportunity for well being will not be achieved.

Beyond this general statement, no simple authoritative definition or list of SDH exists. The European Office of WHO [19] enumerates SDH under topic headings including the social gradient of (dis)advantage, early childhood environment, social exclusion, social support, work, unemployment, food and transport. Although the scope of this inventory is impressive, it mixes categories: for example working conditions, unemployment and access to transport all contribute to the social gradient. Further confusing the issue is the inclusion of stress and addiction, with the former arguably a pathway through which SDH affect physiology and the latter a response to characteristics of the social environment. Finally, some of the discussion is primarily relevant to high-income countries, rather than to the majority of the world's population. Nevertheless, the extent to which items in the WHO Europe list are related to an individual's economic situation and the way in which a society organizes the provision and distribution of economic resources is informative.

Both for this reason and because of the preceding discussion of how global patterns of illness and death are related to economic factors, we do not distinguish between 'economic' and 'social' determinants of health. In addition, we consider health systems as a SDH, for two reasons. Although the entire rationale for a policy focus on SDH is that health is affected by much more than access to health care, access to care is nevertheless crucial in determining health outcomes and often reflects the same distributions of (dis)advantage that characterize other SDH – a point made eloquently in the context of developing and transition economies by Paul Farmer [20]. Further, how health care is financed functions as a SDH. As noted earlier lack of access to publicly funded care can create destructive downward spirals in terms of other SDH when households have to pay large amounts out of pocket for essential services, lose earnings as a result of illness, or both. The importance of this dynamic in a number of Asian countries is emphasized in recent work by van Doorslaer and colleagues [21].

We start from the premise that the processes comprising globalization affect access to SDH by way of multiple pathways, which we describe in the second article in the series. Because of our focus on health equity (or reducing health inequities) and the fact that the effects of globalization on SDH are almost never uniformly distributed across populations, our focus in these articles is on how globalization affects disparities in access to SDH. The 'equity lens' also informs our concentration on what might be described as negative effects of globalization: we presume that disparities in access to SDH lead to deterioration in the health status of those adversely affected, and that when the result is to increase health inequity that deterioration is unacceptable even if offset by positive impacts (e.g. improved health for the well-off) elsewhere in the economy or the society. Stated another way, we regard as *prima facie *undesirable changes in access to SDH that are likely to increase the socioeconomic gradients in health that are observable in all countries, rich and poor alike [22].

The outline of this series is as follows. The remainder of this article identifies and defends a definition of globalization and describes key strategic and methodological issues, emphasizing how and why the special characteristics of globalization as a focus of research on health equity and SDH demand a distinctive perspective and approach. The second article describes a number of key 'clusters' of pathways leading from globalization to equity-relevant changes in SDH. Building on this identification of pathways, the third article provides a generic inventory of potential interventions, based in part on an ongoing program of research on how policies pursued by the G7/G8 countries affect population health outside their borders [23-29]. It then concludes with a few observations about the need for fundamental change in the values that guide industrialized countries' policies toward the much larger, and much poorer, majority of the world's population living outside their borders.

## Globalization and the global marketplace

Globalization is a term with multiple, contested definitions and meanings [30]. Here we adopt a definition of globalization as "a process of greater integration within the world economy through movements of goods and services, capital, technology and (to a lesser extent) labour, which lead increasingly to economic decisions being influenced by global conditions" [31](p. 1) – in other words, to the emergence of a *global marketplace*. This definition does not assume away such phenomena as the increased speed with which information about new treatments, technologies and strategies for health promotion can be diffused, or the opportunities for enhanced political participation and social inclusion that are offered by new, *potentially *widely accessible forms of electronic communication. However, in contrast to simply descriptive accounts of globalization that do not attempt to identify connections among superficially unrelated elements or to assign causal priority to a specific set of drivers (e.g. [32,33]), we adopt the view of Woodward and colleagues that " [e]conomic globalization has been the driving force behind the overall process of globalization over the last two decades" [34](p. 876). This view is supported by evidence that many dimensions and manifestations of globalization that are not at first glance economic in nature are nevertheless best explained with reference to their connections to the global marketplace and to the interests of particular powerful actors in that marketplace. For example, the globalization of culture is inseparable from, and in many instances driven by, the emergence of a network of transnational mass media corporations that dominate not only distribution but also content provision through the allied sports, cultural and consumer product industries [35-37]. Relatedly, global promotion of brands such as Coca-Cola and McDonald's is a cultural phenomenon but also an economic one (driven by the opportunity to expand profits and markets), even as it contributes to the "global production of diet" [38] and resulting rapid increases in obesity and its health consequences in much of the developing world.

The definition of globalization we adopt does not ignore global transmission of ideas and information that are not commercially produced – but here again, reasons exist to focus on economic issues and on the interplay of ideas and interests. Perhaps the most conspicuous illustration of this point is the embrace of 'free' markets and global integration as the only appropriate bases for national macroeconomic policy – a phenomenon that leads us to examine some of the key drivers of globalization, as distinct from the manifestations of globalization processes themselves. To provide historical context, Polanyi's [39] research on the development of markets at the national level showed that markets are not 'natural,' but depend on the creation and maintenance of a complicated infrastructure of laws and institutions. This insight is even more salient at the international level: "It is a dangerous delusion to think of the global economy as some sort of 'natural' system with a logic of its own: It is, and always has been, the outcome of a complex interplay of economic and political relations" [40](p. 3–4). The connection between ideas and economic interests is supplied by the fact that that contemporary globalization has been promoted, facilitated and (sometimes) enforced by political choices about such matters as trade liberalization, financial (de)regulation; provision of support for domestically headquartered corporations [42]; and the conditions under which development assistance is provided. We regard contemporary globalization as having emerged in roughly 1973 with the start of the first oil supply crisis, the resulting impacts on industrialized economies, and the investment of 'petrodollars' in high-risk loans to developing countries that contributed to the early stages of the developing world's debt crises. However, identifying a precise starting point is less important than recognizing that some time in the early 1970s the world economic and geopolitical environment changed decisively, so that (for instance) by 1975 the Trilateral Commission was warning of a "Crisis of Democracy" in the industrialized world [41]. By the mid-1990s, a consortium of social scientists convened to assess the prospects for "sustainable democracy" noted that key Western governments have promoted an "intellectual blueprint ... based on a belief about the virtues of markets and private ownership" with the consequence that: "For the first time in history, capitalism is being adopted as an application of a doctrine, rather than evolving as a historical process of trial and error"[43](p. viii).

The blueprint has been promoted and implemented by national governments both individually and through multilateral institutions like the World Bank, the International Monetary Fund (IMF) and more recently the World Trade Organization [43-46]. Within these institutions, the distribution of power is highly unequal: The G8 nations (the G7 group of industrialized economies plus Russia) "account for 48% of the global economy and 49% of global trade, hold four of the United Nations' five permanent Security Council seats, and boast majority shareholder control over the International Monetary Fund (IMF) and the World Bank" [47]; their influence on World Bank and IMF policies is magnified because some decisions require supermajorities [48](p. 27–8). Networks of academic and professional elites, often with connections to industrialized country governments and institutions like the World Bank and IMF, have likewise played an important role in the outward diffusion of market-oriented ideas about policy design, as shown e.g. by the work of Babb [49] on academic economists in Mexico, Lee & Goodman [50] on the World Bank's role in promoting health sector 'reform', and Brooks [51](p. 54–65) and Mesa-Lago and Müller [52](p. 709–712) on the Bank's role in promoting privatization of public pension systems, especially in Latin America.

To be sure, the diffusion of ideas as an element of globalization involves more than just ideas about markets, and some aspects of the process function as an important counterbalance. Notably, civil society organizations (CSOs) in various policy fields have taken advantage of opportunities for rapid transnational information sharing opened up by advances in computing and telecommunications – the indispensable technological infrastructure of globalization, which cannot be understood in isolation from the needs of its corporate users [53] yet is amenable to use for quite different purposes. Perhaps the best-known illustration of the political influence of CSOs as they relate to health and globalization is their role in challenging the primacy of economic interests as defended by multilateral institutions. In the 1990s, CSO activity contributed to withdrawal from negotiations on a Multilateral Agreement on Investment by the French government, and their subsequent abandonment by the Organization for Economic Cooperation and Development [54]; in the early 2000s, it resulted in an interpretation of the Agreement on Trade-Related aspects of Intellectual Property (TRIPs) that allows health concerns, under some circumstances, to 'trump' the harmonized patent protection that was actively promoted by pharmaceutical firms during the negotiations that led to the establishment of the WTO [55-58]. However, concerns remain about the practical effect of this interpretation because of informal pressures from the pharmaceutical industry and industrialized country governments and 'TRIPs-plus' provisions in bilateral trade agreements, and one academic observer is sceptical about the extent to which intellectual property protection has created barriers to access to essential medicines [59].

Some women's health movements, as another example, have become "transnationalized," partly within, and shaping the agenda of, the institutional framework provided by the UN system [60]. CSOs have also been important actors in the admittedly uneven and incomplete international diffusion of human rights norms in the decades following the 1948 Universal Declaration of Human Rights – norms to which we return in the third article as a potential challenge to the current organization of the global marketplace. Thus, although we insist on the primacy of the economic dimensions of globalization, and on the economic elements of SDH, our view is not narrowly deterministic, and allows for the possibility of effective challenges to the interests that dominate today's global economic and political order.

## Globalization and social determinants of health: Recent conceptual milestones

As background to a discussion of research methods and strategies, it is worthwhile to provide a selective overview of previous conceptual milestones that have contributed to understanding the influences on SDH. A 1987 UNICEF publication on *Adjustment with a Human Face *[61] reported early and important findings on how what we would now call globalization was affecting SDH. The study involved 10 countries (Botswana, Brazil, Chile, Ghana, Jamaica, Peru, Philippines, South Korea, Sri Lanka, Zimbabwe) that had adopted policies of domestic economic adjustment in response to economic crises that led them to rely on loans from the IMF – a dynamic that is described in the second article of the series. In many cases the policies adopted had resulted in deterioration in key indicators of child health (e.g. infant mortality, child survival, malnutrition, educational status) and in access to SDH (e.g. availability and use of food and social services), with reductions in government expenditure on basic services emerging as a key intervening variable. The study situated these national cases within an analytical framework that linked changes in government policies (e.g. expenditures on education, food subsidies, health, water, sewage, housing and child care services) with selected economic determinants of health at the household level (e.g. food prices, household income, mothers' time) and selected indicators of child welfare [62]. Based on that analysis, the study identified a generic package of policies that would minimize the negative effects of economic adjustment by protecting the basic incomes, living standards, health and nutrition of the poor or otherwise vulnerable [63] – priorities that have similarly been stressed in subsequent policy analyses. However, in the context of globalization an important limitation is that only the final chapter of the UNICEF study [64] addressed elements of the international policy environment that might facilitate implementation of "adjustment with a human face" in some countries while obstructing it in others, and the study as a whole did not directly address the comparative merits of "compensating for adjustment" [65] in health policies and programs and rethinking the adjustment process itself.

**Figure 1:**
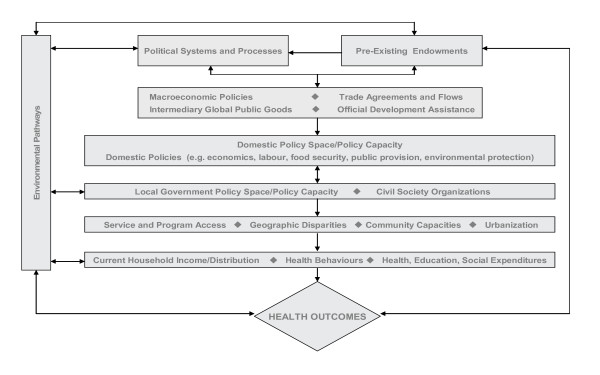
**Globalization and Health: Simplified Pathways and Elements**. Source: [66].

In work for WHO, Woodward and colleagues [34] devised an explanatory model that focused on "five key linkages from globalization to health," three direct and two indirect. Direct effects included impacts on health systems, health policies, and exposure to certain kinds of hazards such as infectious disease and tobacco marketing; indirect effects were those "operating through the national economy on the health sector (e.g. effects of trade liberalization and financial flows on the availability of resources for public expenditure on health, and on the cost of inputs); and on population risks (particularly the effects on nutrition and living conditions resulting from impacts on household income)." Here, again, we see an emphasis on the economic aspects both of globalization and of SDH. This model has the advantage of focusing on the range of policy choices (by both governmental and private actors) that operate at the supranational level to affect health, while being limited in its focus primarily on health systems relative to other SDH. A subsequent WHO-supported systematic review examined numerous models of the relations between globalization and health, generating a diagrammatic synthesis hierarchically organized around various levels of analysis ranging from the supranational to the household [66,67] (Figure 1). Key strengths of this synthesis are its recognition of the importance of environmental pathways (reflected in the discussion of this topic in the second article in the series); its attention to how globalization influences the context within which national and subnational govenrnments make and implement policy; and its acknowledgment of the role of political systems and processes and pre-existing endowments (natural resources, geographic location, levels of education) as mediators of that influence. Conversely, a limitation is a lack of focus on the specific pathways that lead to changes in individual and population health status by way of SDH.

**Figure 2:**
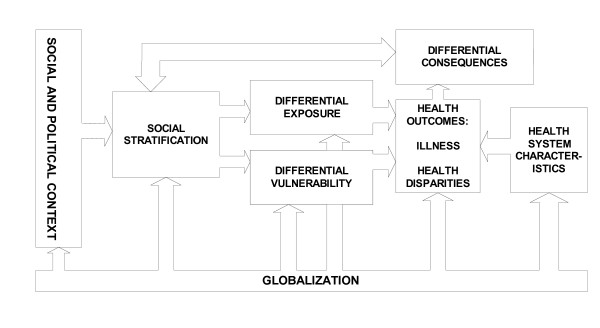
**Globalization and Health: A Framework for Analysis**. Source: Modified from [68] by the authors.

In a conceptual framework developed specifically for analyzing those pathways, Diderichsen and colleagues [68](p. 14) identify "four main mechanisms – social stratification, differential exposure, differential susceptibility, and differential consequences – that play a role in generating health inequities." Globalization can affect health outcomes by way of each of these mechanisms, and the authors' reference to the influence on stratification of "those central engines in society that generate and distribute power, wealth and risks" [68](p. 16) is especially apposite in this context. A variant of this model was provisionally adopted as an organizing framework in a concept paper for the Commission on Social Determinants of Health [69], and has been further modified for purposes of the Globalization Knowledge Network (Figure 2 presents the model in simplified form).

A stylized example shows the model's relevance. Import liberalization may reduce the incomes of some workers in sectors serving the domestic market, or shift them into the informal economy, thereby affecting social stratification, differential exposure (e.g. as workers are exposed to new hazards) and differential vulnerability (e.g. as income loss means adequate nutrition or essential health care become harder to afford, or in the extreme cases in which women are driven to reliance on "survival sex" [70,71]). Increased vulnerability may also magnify the negative consequences of ill health by reducing the resources available to households to pay for health care or absorb earnings losses, increasing the chance of falling into "poverty traps" (hence the feedback loop to social stratification). Import liberalization may also reduce tariff revenues (and therefore funds available for public expenditures on income support or health care) in advance of any offsetting increases from income and consumption taxes. In countries with high levels of external debt, the need to conserve funds for repaying external creditors, perhaps by initiating or increasing user fees for health and education, may create a further constraint. (The rationale for including health systems as a separate element of the diagram now becomes apparent.) Conversely, if import liberalization is matched by improved access to export markets, new employment opportunities may be created for specific groups, such as women working in export processing zones, who are thereby empowered to escape patriarchal social structures (social stratification) and reduce their economic vulnerability.

## Methodological issues

Despite the sense of simplicity created by diagrammatic representations, no single such representation will be adequate to capture the complexities of globalization and its influences in more than a limited number of situations. Globalization comprises multiple, interacting policy dynamics or processes the effects of which may be difficult if not impossible to separate. Pathways from globalization to changes in SDH are not always linear, do not operate in isolation from one another, and may involve multiple stages and feedback loops. Similarities exist with the task of analyzing causal links between environmental change and human health, which "are complex because often they are indirect, displaced in space and time, and dependent on a number of modifying forces," in the words of WHO's synthesis of the health implications of the findings of the Millennium Ecosystem Assessment project [72] (p. 2).

It is therefore necessary to rely on evidence generated by multiple disciplines, research designs and methodologies – the approach now widely described as transdisciplinary [73] – comprising both qualitative and quantitative findings. Issues of scale are also relevant: for example, research that situates data from local-scale survey research in the context of structural adjustment in Zimbabwe [74,75] and that identifies globalization-related influences on health in South Africa [76] demonstrates the need to integrate work using different units of analysis (e.g. the household, the region, the national economy) in order to describe relevant mechanisms of action in sufficient detail, and to reflect intra-national disparities (e.g. by region, class and gender) that are not apparent from national level data [77-79].

The evidence base for assessing globalization's effects on SDH and identifying opportunities for intervention is therefore different from, and more heterogeneous than, the body of research that is available with respect to clinical and (many) public health interventions. Notably, qualitative research provides information about differential impacts (e.g. by region, gender, kind of employment) that are not revealed by standard indicators, and about such matters as the problems created by the imposition of user charges and cost recovery in water and sanitation systems [80]. Within the ethnographic literature, Schoepf [81-84] demonstrates the value of qualitative evidence about the relations between micro-level outcomes and such macro-level factors as falling commodity prices, domestic austerity policies that involved cuts in public sector employment and in subsidized access to health care, and migration driven by economic desperation. For further illustrations of the value of qualitative research see e.g. the World Bank's Voices of the Poor study [85,86]; the report of the Structural Adjustment Participatory Review International Network [87]; and a summary of studies of sources of livelihood in KwaZulu-Natal, South Africa by Lund [88].

Policy-relevant linkages between globalization and SDH are therefore best described, and the strength of evidence evaluated, by way of syntheses that incorporate several elements, including (but not limited to): (a) description of the national and international policy context and its history; (b) country- or region-specific studies that describe changes in determinants of health, such as the level and composition of household income, labour market changes, access to education and health services; (c) evidence from clinical and epidemiological studies that relates to demonstrated or probable changes in health outcomes arising from those impacts; (d) ethnographic research, field observations, and other first-hand accounts of experience 'on the ground'. This choice of elements is not random; it recognizes the need for study at the various levels identified in Figure 1, and the need not only to connect contextual factors with changes in SDH and their distribution, but also to demonstrate where feasible a relation between changes in SDH and changes in health outcomes.

At the same time, the complexity of the evidence base and the relevant causal chains means that rarely will it be possible to state conclusions with the degree of conclusiveness that may be possible in a laboratory situation or even in many epidemiological study designs, where almost all variables can be controlled. In the words of social epidemiologist Michael Marmot, who now chairs the CSDH: "The further upstream we go in our search for causes," and globalization is the quintessential upstream variable, the greater the need to rely on "observational evidence and judgment in formulating policies to reduce inequalities in health" [89](p. 308). The choice and defence of a standard of proof – how much evidence is enough – is also important. As in the context of national public health and regulatory policy [90,91], the decision must be made with explicit reference to the underlying, potentially competing values. Excessive concern with avoiding false positive findings (Type I errors, or the incorrect rejection of the null hypothesis) can supply, as in other contexts, a credible and convenient rationale for doing nothing. This is the "tobacco industry standard of proof" [92](pp. 66–67) – so demanding that there is always room to claim that evidence is less than conclusive. In the environmental policy context, Page [90] has convincingly demonstrated the negative health outcomes that may result when standards of proof are set without explicit reference to the possible consequences of being wrong in different kinds of ways. On this point, it cannot be emphasized too strongly that the choice of a standard of proof is inescapably value-driven, and is not always a choice with respect to which scientific researchers have any special competence.

In a study that illustrates application of the preceding insights about explanation, De Vogli and Birbeck [93] identify five multi-step pathways that lead from globalization to increased vulnerability to HIV infection and its consequences among women and children in sub-Saharan Africa by way of: currency devaluations, privatization, financial and trade liberalization, implementation of user charges for health services and implementation of user charges for education. The first two pathways operate by way of reducing women's access to basic needs, either because of rising prices or reduced opportunities for waged employment. The third operates by way of increasing migration to urban areas, which simultaneously may reduce women's access to basic needs and increase their exposure to risky consensual sex. The fourth pathway (health user fees) reduces both women's and youth's access to HIV-related services, and the fifth (education user fees) increases vulnerability to risky consensual sex, commercial sex and sexual abuse by reducing access to education. The explanatory approach adopted is congruent with recent reviews of research on HIV/AIDS, tuberculosis and malaria [3,4] which concluded that vulnerability to all three diseases is closely linked; that poverty, gender inequality, development policy and health sector 'reforms' that involve user fees and reduced access to care are important determinants of vulnerability; and that " [c]omplicated interactions between these factors, many of which lie outside the health sector, make unravelling of their individual roles and therefore appropriate targeting of interventions difficult" [4](p. 268).

A choice must also be made about the time frame of concern. In the long run wealthier societies are healthier, albeit with wide variations in health status at a given level of income per capita [94,95]. It can be argued that the optimal, or at least most realistic, approach to improving SDH is the one that will maximize economic growth in the countries or regions of concern, even at the cost of substantial short-term deteriorations in health status or increases in health disparities. This argument is implicit in a widely cited article claiming that "Globalization is good for your health, mostly,"[96] and was stated explicitly by a team of World Bank economists with respect to the transition economies of the former Soviet bloc [97]. However, the empirical uncertainties associated with this position lead Angus Deaton, one of the leading researchers on the relations between economic growth and health, to warn flatly that "economic growth, by itself, will not be enough to improve population health, at least in any acceptable time." [98] The issue of acceptable time raises the ethical question of how long is too long. As suggested by Deaton, diffusion of the benefits of economic growth in ways that lead to widespread improvements in population health is neither automatic nor rapid: it took more than 50 years in the industrial cities of nineteenth-century England, for example [99-101]. Given the frequency with which globalization has resulted in deterioration in SDH for substantial segments of national populations, despite impressive economic growth as measured by national indicators, this is not just an academic point. We return to it in the third article in the series.

## Competing interests

The author(s) declare that they have no competing interests.

## Authors' contributions

The authors contributed equally to the conception and design of the study; acquisition, analysis and interpretation of data; and drafting of the manuscript. Both authors have read and approved the final manuscript.
